# Retrovirus-like particles in EBV-negative Burkitt's lymphoma cell line but not in EBV-DNA-positive lines from patients with ataxia telangiectasia and Down's syndrome.

**DOI:** 10.1038/bjc.1979.74

**Published:** 1979-04

**Authors:** M. Kotler, H. Balabanova, A. Friedmann, Y. Becker

## Abstract

**Images:**


*Br. J. Cancer* (1979) **39**, 414

### RETROVIRUS-LIKE PARTICLES IN EBV-NEGATIVE BURKITT'S LYMPHOMA CELL LINE BUT NOT IN EBV-DNA-POSITIVE LINES FROM PATIENTS WITH ATAXIA TELANGIECTASIA AND DOWN'S SYNDROME

M. KOTLER, H. BALABANOVA, A. FRIEDMANN* AND Y. BECKER

*Laboratory for Molecular Virology, Hebrew University-Hadassah Medical School and *Department of Genetics, The Hebrew University of Jerusalem, Israel*

Received 1 August 1978 Accepted 7 December 1978

**Summary.—Retrovirus-like particles can be recovered by arginine deprivation from the BJAB-1 Epstein-Barr virus (EBV) negative cell line derived from an African patient with typical Burkitt's lymphoma. These particles resemble retroviruses in their morphology and in their physicochemical properties. Particles with a similar morphology were obtained from derivative cell lines established by infecting BJAB-1 cells with EBV. On the other hand, retrovirus-like particles could not be induced in EBV-DNA-positive lymphoblastoid cell lines derived from non-leukaemic patients with ataxia telangiectasia and Down's syndrome and from a patient with infectious mononucleosis.**

HUMAN lymphoid cells derived from lymphoma and leukaemia patients were found to contain retrovirus genetic information and to induce the formation of type C virus particles under suitable conditions (Gallagher & Gallo, 1975; Kotler *et al.*, 1975; Mak *et al.*, 1974; Nooter *et al.*, 1975). Initial studies by Kotler *et al.* (1973, 1975) showed that cultured lymphoblastoid cell lines from patients with leukaemia and Burkitt's lymphoma produce retrovirus-like particles after incubation in arginine-deficient medium. These particles contain an endogenous reverse-transcriptase activity that is stimulated by the addition of exogenous templates such as oligo(dG).poly(rC) or oligo(dT).poly(rA). A similar observation on retrovirus particles released from the QIMR-WIL cell line was reported by Klucis *et al.* (1976).

More recently, Kotler *et al.* (1977) reported that the arginine-deprived lymphoblastoid cell lines, Raji and P3HR-1, contain neoantigens that cross-react with simian sarcoma and Rauscher mouse leukaemia virus antigens. These cell lines are EBV-DNA positive. It was therefore of interest to determine whether C-type particles could also be induced in the BJAB-1 EBV-DNA-negative lymphoblastoid cell line derived from a patient with typical African Burkitt's lymphoma (Menezes *et al.*, 1975). Infection of BJAB-1 cells with EBV from marmoset lymphoblasts (Miller *et al.*, 1972) converted them into EBV-DNA-positive cells, and enhanced their ability to proliferate *in vitro* (Steinitz & Klein, 1975). As controls we used lymphoblastoid cell lines derived from a patient with infectious mononucleosis, and lines which developed spontaneously from blood samples of non-leukaemic persons with ataxia telangiectasia (AT) and Down's syndrome. These cell lines contain EBV DNA and EBV nuclear antigen (Cohen *et al.*, 1978). Patients with AT and Down's syndrome have a higher incidence of leukaemias and lymphomas

Correspondence to: Professor Y. Becker, Laboratory for Molecular Virology, Hebrew University-Hadassah Medical School, Jerusalem.

than the normal population (Hecht & McCaw, 1977; Lubiniecki, 1977).

In this study we demonstrate that after arginine deprivation, BJAB-1 cells as well as EBV-infected sublines, release retrovirus-like particles that are biophysically and morphologically similar to human and monkey viruses described in recent years. The particles that appear after arginine deprivation have a density of 1·16–1·17 g/ml in sucrose gradients and differ from the particles described by Smith *et al.* (1976) that sediment at 1·18–1·22 g/ml. Lymphoblastoid cell lines derived from non-leukaemic persons with genetic disorders or infectious mononucleosis did not produce retrovirus-like particles after arginine deprivation.

### MATERIALS AND METHODS

*Cell lines.*—The EBV-DNA-negative BJAB-1 cell line (Menezes *et al.*, 1975), derived from lymphoblasts of an African patient with Burkitt's lymphoma, and the GC/BJAB-1 and AW/BJAB-1 cell lines, were kindly supplied by Professor George Klein, Karolinska Institute, Stockholm. The GC/BJAB-1 and AW/BJAB-1 sublines, that are EBV-DNA positive and produce the virus (Steinitz & Klein, 1975; Klein *et al.*, 1975; Clements *et al.*, 1975) resulted from infection of BJAB-1 cells with EBV from the B95-8 marmoset cell line (Miller *et al.*, 1972). These cells were grown in RPMI 1640 medium (Grand Island Biological Co.) containing 10% heat-inactivated foetal calf serum (GIBCO) as suspension cultures in 250 ml glass bottles at an initial concentration of $4–5 \times 10^5$ cells/ml. The medium for BJAB-1 cells was supplemented with 24 u/l of insulin (Nordisk Insullaboratorium, Copenhagen) since insulin stimulated the growth rate of BJAB-1 cells. Every 3–4 days the cells were subcultured.

Lymphoblastoid cell lines from 3 patients with AT and 2 with Down's syndrome were used as controls. These cell lines were found to contain EBV DNA and EBV nuclear antigens, and one line produced EB virus (Cohen *et al.*, 1978). One cell line was derived from a patient with infectious mononucleosis. These lines were obtained from Professor M. M. Cohen, Department of Human Genetics, Hadassah Medical Centre, Jerusalem. The cells were propagated in RPMI 1640 medium containing 25% foetal calf serum.

Arginine deprivation was carried out by incubating the cell cultures at 37°C for 24 h in the presence of insulin, in medium lacking arginine and containing 2% dialysed foetal calf serum.

*Virus purification.*—Cell debris was removed from the medium by centrifugation for 10 min at 9000 *g* in a Sorvall centrifuge. The supernatant fluids were then centrifuged for 60 min at 25,000 rev/min in the Beckman rotor 30 or 52 Ti. The pellets were suspended in RPMI medium to about 1/200th of the starting volume. The concentrated preparations were centrifuged in 15% (w/v) sucrose layered on to a 65% (w/v) sucrose cushion prepared in TE buffer (10 mM Tris.HCl, 1 mM EDTA, pH 8·0) for 60 min at 45,000 rev/min in the Beckman SW 50·1 rotor. The particles banding at the top of the 65%-sucrose cushion were diluted in buffer and centrifuged at 45,000 rev/min for 180 min using the same rotor. The gradients were collected dropwise and the density of selected fractions was determined.

*RNA-dependent DNA polymerase assay.*—Virus samples were suspended in buffer to a final concentration of 0·01M Tris.HCl, pH 7·8, 0·001M EDTA and 0·02% (v/v) Nonidet P-40 (NP-40). The virus suspension was mixed with the reaction mixture to a final concentration of 0·1 mM dGTP, dCTP and dATP (Sigma, St Louis, Mo.) and 125 $\mu$Ci of [$^3$H]-TTP (sp. act. 50 Ci/mmol, The Radiochemical Centre, Amersham, England), 10 mM MgCl and 20 mM KCl.

*Exogenous reaction.*—The virus samples were treated with the same buffer used for the endogenous reactions, and the reaction mixture contained 50 mM Tris.HCl, pH 7·8, 100 mM KCl, 2 mM dithiothreitol, 13 $\mu$M dGTP, 13 $\mu$M TTP, 3 $\mu$M [$^3$H]-dGTP (sp. act. 23 Ci/mM, The Radiochemical Centre, Amersham) and 25 $\mu$g/ml of oligo (dG).poly(rC).

*Preparation of cells for electron microscopy.*—The cells were washed with Tyrode's buffer, centrifuged gently into a pellet and fixed in the cold for 60 min in 1% glutaraldehyde. After washing with Sorenson's buffer, the cells were fixed for 30 min in 1% osmium tetroxide, dehydrated and embedded in epoxy resin (Epon 812). Thin sections were prepared, stained with uranyl acetate and lead citrate, before examination in a JEM 7A electron microscope.

### RESULTS

*Virus-like particles released from BJAB-*1 *cells incubated in arginine-deficient medium*

The BJAB-1 lymphoblastoid cell line was incubated in RPMI 1640 medium lacking arginine for 24 h. The medium was clarified and the particulate material was concentrated and analysed in sucrose gradients (Fig. 1A). Enzyme activity was detectable in the 1·20 and 1·18 g/ml regions of the gradient, with a distinct peak in the 1·16 g/ml region. Incubation of the isolated virus-like particles from the 1·16 g/ml region with the exogenous primer template oligo(dG).poly(rC) led to incorporation of [$^3$H]-dGTP, but not of [$^3$H]-TTP into polymeric form (Table). A similar activity was found using 0·8 mM $Mn^{++}$ instead of 10 mM $Mg^{++}$ in the reaction mixture. Such activity was not found in the high-density fractions (1·20 g/ml, Table). These experiments, which were repeated several times, led us to conclude that the isolated particles from arginine-deficient cultures contain a reverse-transcriptase activity and resemble the virus particles released from P3HR-1, Raji and 1301 lymphoblast cell lines used as controls (Table).

In control experiments with particulate material from arginine-containing medium of BJAB-1 cell cultures, the DNA-polymerase activity was found in the 1·17–1·22 g/ml region of the gradient (Fig. 1B). This activity was neither sensitive to RNase nor stimulated by the addition of exogenous template oligo(dG).-poly(rC) (not shown,) and thus differed from the reverse transcriptase activity of retroviruses. A similar activity was described in media harvested from cultured human leukaemia cells by Smith *et al.* (1976).

*Electron microscopy of BJAB-*1 *cell sections.*—BJAB-1 cells incubated in an arginine-deficient medium were harvested, sectioned and examined by electron microscopy. In every preparation, virus-like particles were detected in the extracellular spaces (Fig. 2A, B) or budding from the cell membrane (Fig. 2C, D). The virus-like particles have a dense core

FIG. 1.—DNA-polymerase distribution after centrifugation in sucrose gradients of the particulate material released into the medium of arginine-deprived (A) and undeprived (B) BJAB-1 cells. The particulate material was centrifuged for 180 min at 45,000 rev/min in the SW 50.1 Beckman rotor at 4°C; gradients were fractionated dropwise and aliquots from each fraction were tested for the presence of endogenous DNA-polymerase activity in a total volume of 25 µl per reaction. The incubation was at 37°C for 30 min, and the reaction was stopped by the addition of cold TCA.

TABLE.—*DNA-polymerase activity in virus-like particles isolated in density sucrose gradients*

| Virus particles from cell line | Density region (g/ml) | Reverse transcriptase: Oligo(dG) . poly(rC) +[$^3$H]-dGTP (ct/min) | Reverse transcriptase: Oligo(dG) . poly(rC) +[$^3$H]-TTP (ct/min) | Reverse transcriptase: No template [$^3$H]-dGTP (ct/min) |
|---|---|---|---|---|
| BJAB-1 | 1·16 | 2200 | 100 | 300 |
| BJAB-1 | 1·20 | 1900 | 1600 | 2200 |
| P3HR-1* | 1·17 | 11000 | 2000 | ND** |
| Raji* | 1·16 | 5726 | ND | 1994 |
| 1301* | 1·17 | 10000 | 1200 | 2500 |

Particulate material from medium lacking arginine of human lymphoma cell lines was analysed in sucrose gradients as described in the legend to Fig. 1. Aliquots of 10 μl from each fraction were tested for exogenous activity in a total volume 25 μl with incubation at 37°C for 20 min. The reactions were stopped by the addition of cold trichloroacetic acid (TCA). Each fraction was tested in the presence of oligo(dG).poly(rC) and [$^3$H]-dGTP for reverse transcriptase, in the presence of template plus [$^3$H]-TTP to distinguish the polymerase activity from the terminal transferase and in the absence of template for nonspecific activity.

* The P3HR-1 and Raji cell lines from Burkitt's lymphoma and 1301 line from human leukaemia, previously described by Kotler *et al.* (1975, 1977) were used as controls.

**ND—not done.

(Fig. 2B) but many have an irregular shape, and in some of them the core is acentric (Fig. 2A) and may vary in size (Fig. 2A, B). Budding particles can be detected in many cells, as shown in Fig. 2C and 2D. All attempts by electron microscopy to reveal virus-like particles in sections of BJAB-1 cells grown in complete medium yielded negative results.

### *Virus-like particles released from* in vitro *infected BJAB-1 cells*

BJAB-1 cells infected with EBV harvested from the B95-8 marmoset cell line produced sublines GC/BJAB and AW/BJAB that contained EBV DNA in their nuclei. Electron microscopy of GC/BJAB-1 lymphoblasts after 24 h incubation in an arginine-deficient medium revealed virus-like particles budding through the cell membrane (Fig. 3A, B) or free in the extracellular spaces (Fig. 3C). The budding virus-like particles have a large core, similar to that of the virus particles released from BJAB-1 lymphoblasts (Fig. 3D). The released virus-like particles have a large, somewhat irregular core with a dense central area (Fig. 3C). These results indicate that retrovirus-like particles can be detected after arginine deprivation irrespective of the presence or absence of EBV genomes.

Incubation of the BJAB-1 lymphoblastoid subline AW/BJAB in an arginine-deficient medium produced the release of virus-like particles into the medium (Fig. 4A, B). These virus-like particles have a distorted structure, but the large core is distinct and similar to the virus-like particles from BJAB-1 and GC/BJAB cells. Similar odd-looking virus-like particles were found in another cell line which resulted from fusion of BJAB-1 cells with EBV-DNA-positive Raji lymphoblasts (Fig. 4C). These cells did not release virus-like particles when grown in complete medium.

### *Lack of retroviruses in arginine-deprived non-leukaemic lymphoblastoid cells*

It was of interest to study the response to arginine deprivation of "B" type lymphoblastoid cells that developed spontaneously from the blood of individuals with two types of genetic disorders, ataxia telangiectasia (AT) and Down's syndrome (trisomy 21). A lymphoblastoid cell line from a patient with infectious mononucleosis was also investigated. These cell lines contain EBV DNA and EBV nuclear

FIG. 2.—Electron micrographs of thin sections prepared from BJAB-1 cells grown in arginine-deprived medium. A. Part of a cell showing the nucleus and cytoplasm and retrovirus-like particles in the extracellular space. Note the irregular shape of the particles and the acentric cores in some of them. B. An enlarged particle showing the morphology of the core. C. and D. Particles budding from the cell surface.

FIG. 3.—Electron micrograph of thin sections prepared from EBV-infected BJAB-1 cells (GC/BJAB-1) grown in arginine-deprived medium. A. Enlarged particle showing doughnut-like core. B. A budding particle. C. Particles found in extracellular spaces.

FIG. 4.—Electron micrographs of thin sections prepared from EBV-infected BJAB-1 cells (A,B) termed AW/BJAB-1 (similar to the cell line in Fig. 3) and a line derived from fusion of BJAB-1 cells with Raji lymphoblasts (C). Note the irregularity and fragility of the particle membranes.

antigens, and each represents a clone from a single EBV-infected lymphocyte. In two separate experiments, no retrovirus-like particles were observed by electron microscopy in any of these cell lines, whether grown in the absence or presence of arginine. This result indicates that the lymphoblastoid cells do not contain a retrovirus which is inducible by arginine deficiency.

### DISCUSSION

This study deals with the induction of virus-like particles from the EBV-DNA-negative BJAB-1 lymphoblastoid cell line obtained from an African patient with Burkitt's lymphoma. Electron-microscopic analysis of BJAB-1, and derivative sub-lines infected with EBV, revealed the presence of retrovirus-like particles. This was concluded from virus morphology, the presence of budding virus particles and reverse-transcriptase activity in the 1·16–1·17 g/ml region in sucrose gradients. The BJAB-1 virus particles differ in their morphological appearance from the type C particles induced in Raji and P3HR-1 cells (Kotler *et al.*, 1975, 1977).

Morphologically similar virus particles were also obtained from sublines of BJAB-1 lymphoblasts which were infected *in vitro* with EBV. In addition to containing EBV DNA, the BJAB-1-derived cell lines (GC/BJAB and AW/BJAB) retained their ability to produce retrovirus particles. In this respect the GC/BJAB lymphoblasts resemble Raji and P3HR-1 lymphoblasts, which carry EBV DNA in their nuclei and are capable of synthesizing a retrovirus when incubated in an arginine-deficient medium (Kotler *et al.*, 1975, 1977). The presence of EBV DNA in the nuclei of Burkitt lymphoblasts does not affect the ability of the cells to express the genetic information of a latent virus under certain conditions.

The morphology of the viruses demonstrated in BJAB-1 and derivative cell lines distinguishes them from the known B and C-type retrovirus groups. The mode of budding and the acentric location of the core distinguishes these particles from C-type particles. There is some resemblance to B-type particles, but there are differences in the surface projections and in the morphology of the mature particles. Particles similar to those described here were found in HeLa cells (Gelderblom *et al.*, 1972) and in human neoplastic haemopoietic tissues (Popovic *et al.*, 1974).

The virus particles produced by GC/BJAB-1 cells, although morphologically similar to BJAB-1 virus particles, pose another problem. The GC/BJAB lymphoblasts were derived from BJAB-1 cells infected with EBV from B95-8 marmoset lymphoblasts which produce EBV (Miller *et al.* 1972). It is possible that the B95-8 lymphoblasts contain or produce a marmoset endogenous retrovirus that may have been present in the EBV preparation used to infect the human BJAB-1 lymphoblasts. Preliminary electron microscopic studies on B95-8 lymphoblasts incubated in arginine-free medium revealed retrovirus-like particles in the marmoset cell. Laboratory contamination of the BJAB-1 cells by a retrovirus can be ruled out for the following reasons: (a) The retroviruses were found in the BJAB-1 cells only after arginine deprivation, not under regular conditions of growth, (b) the morphology of the particles differed from that of type-B and C viruses, but it resembled that of some primate viruses (Chopra *et al.*, 1972) which have never been grown in our laboratory, and (c) the particles found in the Ramos cell line which had been transferred in a nude mouse (Kotler *et al.*, 1977) differed entirely from the viruses found in the BJAB-1 and derivative cell lines.

The lymphoblastoid cell lines from patients with AT, Down's syndrome and infectious mononucleosis contain EB virus genetic information, but did not respond with retrovirus production when incubated in an arginine-deficient medium. The cell lines from persons with autosomal recessive genetic syndromes were chosen since leukaemia proneness of such individuals is higher than in the general population (Hecht & McCaw, 1977; Lubiniecki, 1977). It is possible that lymphoblastoid cells from non-leukaemic patients with AT and Down's syndrome behave as normal cells in respect of the absence of retrovirus genetic information in the cells. When leukaemia or lymphoma develops in a person with a genetic disorder, the tumour cells may be derived from a lymphocyte infected *in vivo* with a retrovirus. Lymphoblastoid cells from an AT patient with leukaemia should therefore contain retrovirus genetic information.

We would like to thank Dr Robin Weiss, Imperial Cancer Research Fund, London, for useful discussions, and also Professor George Klein, Karolinska Institute, Stockholm, and Professor M. M. Cohen, Hadassah Hospital, Jerusalem, for the cell lines and for helpful suggestions. Our thanks to Dr Julia Hadar for assistance with the manuscript. This study was supported by a grant from the Leukemia Research Foundation, Chicago, Illinois, and the Israel Cancer Association.

### REFERENCES

CHOPRA, H. C., HOOKS, J. J., WALLING, M. J. & GIBBS, C. J. (1972) Morphology of simian foamy viruses, with particular reference to virus isolated from spontaneous tumor of a Rhesus monkey. *J. Natl. Cancer Inst.*, **48**, 451.

CLEMENTS, G. B., KLEIN, G. & POVEY, S. (1975) Production of EBV infection of an EBNA-positive subline from an EBNA-negative human lymphoma cell line without detectable EBV DNA. *Int. J. Cancer*, **16**, 125.

COHEN, M. M., SAGI, M., BEN-ZUR, Z. & 4 others (1978) Ataxia telangiectasia: chromosomal stability in continuous lympho-blastoid cell lines. *Cytogenet. Cell Genet.*, **23**, 44.

GALLAGHER, R. E. & GALLO, R. C. (1975) Type-C RNA tumor virus isolated from cultured human acute myelogenous leukemic cells. *Science, N.Y.*, **187**, 350.

GELDERBLOM, H., BAUER, H., OGURA, H., WIGAND, R. & FISHER, A. (1972) Detection of oncornavirus-like particles in HeLa cells. I. Fine structure and comparative morphological classification. *Int. J. Cancer*, **13**, 246.

HECHT, F. & MCCAW, B. (1977) Chromosome instability syndrome. In *Genetics of Human Cancer.* Eds. J. J. Mulvihill, R. W. Miller & J. F. Fraumeni Jr. New York: Raven Press. p. 105.

KLEIN, G., GIOVANELLA, B., WESTMAN, A., STEHLIN, J. S. & MUMFORD, P. (1975) An EBV-genome negative cell line established from an American Burkitt lymphoma; receptor characteristics, EBV-infectibility and permanent conversion into EBV positive sublines by *in vitro* infection. *Intervirology*, **5**, 319.

KLUCIS, E., JACKSON, L. & PARSONS, P. G. (1976) Survey of human lymphoblastoid cell lines and primary cultures of normal and leukaemic leukocytes for oncornavirus production. *Int. J. Cancer*, **18**, 413.

KOTLER, M., BALABANOVA, H., BEN-MOYAL, Z., FRIEDMANN, A. & BECKER, Y. (1977) Properties of the oncornavirus particles isolated from P3HR-1 and Raji human lymphoblastoid cell lines. *Israel J. Med. Sci.*, **13**, 740.

KOTLER, M., BALABANOVA, H., WEINBERG, E., FRIEDMANN, A. & BECKER, Y. (1975) Oncornavirus-like particles released from arginine deprived human lymphoblastoid cell lines. *Proc. Natl Acad. Sci. U.S.A.*, **72**, 4592.

KOTLER, M., WEINBERG, E., HASPEL, O., OLSHEVSKY, U. & BECKER, Y. (1973) Particles released from arginine deprived human leukaemic cells. *Nature (New Biol.)*, **244**, 197.

LUBINIECKI, A. S. (1977) Target theory applied to acute leukemia in Down's syndrome and the general population. In *Genetics of Human Cancer.* Eds. J. J. Mulvihill, R. W. Miller & J. F. Fraumeni, Jr. New York: Raven Press. p. 83.

MAK, N. W., MANASTER, J., HOWATSON, A. F., MCCULLOCH, E. A. & TILL, J. E. (1974) Particles with characteristics of leukoviruses in cultures of marrow cells from leukemic patients in remission and relapse. *Proc. Natl Acad. Sci. U.S.A.*, **71**, 4336.

MENEZES, J., LEIBOLD, W., KLEIN, G. & CLEMENTS, G. (1975) Establishment and characterization of an Epstein-Barr virus (EBV)-negative lymphoblastoid B cell line from an exceptional EBV genome negative African Burkitt's type lymphoma. *Biomedicine*, **22**, 276.

MILLER, G., SHOPE, T., LISCO, H. & LIPMAN, M. (1972) Epstein-Barr virus: Transformation cytopathic changes and viral antigens in squirrel monkeys and marmoset leukocytes. *Proc. Natl Acad. Sci. U.S.A.*, **69**, 383.

NOOTER, K., AARSSEN, A. M., BENTVELZEN, P., DE GROOT, F. G. & VAN PELT, F. G. (1975) Isolation of infectious C-type oncornavirus from human leukaemic bone marrow cells. *Nature, Lond.*, **256**, 595.

POPOVIC, M., PONTEN, J., GROFOVA, M., NILSSON, K., MATOSKA, J. & BENGTSSON, A. (1974) Detection of oncornavirus-like particles in human cell lines derived from tumours of hemopoietic tissue. *Neoplasma*, **21**, 619.

SMITH, C. C., MAVERAKIS, N. H., & ACKERMAN W. W. (1976) Characterization of extracellular particles released from continuous cell cultures derived from human leukemia. *Proc. Soc. Exp. Biol. Med.*, **152**, 645.

STEINITZ, M. & KLEIN, G. (1975) Comparison between growth characteristics of an Epstein-Barr virus (EBV)-genome negative lymphoma line and its EBV converted subline *in vitro*. *Proc. Natl Acad. Sci. U.S.A.*, **72**, 3518.


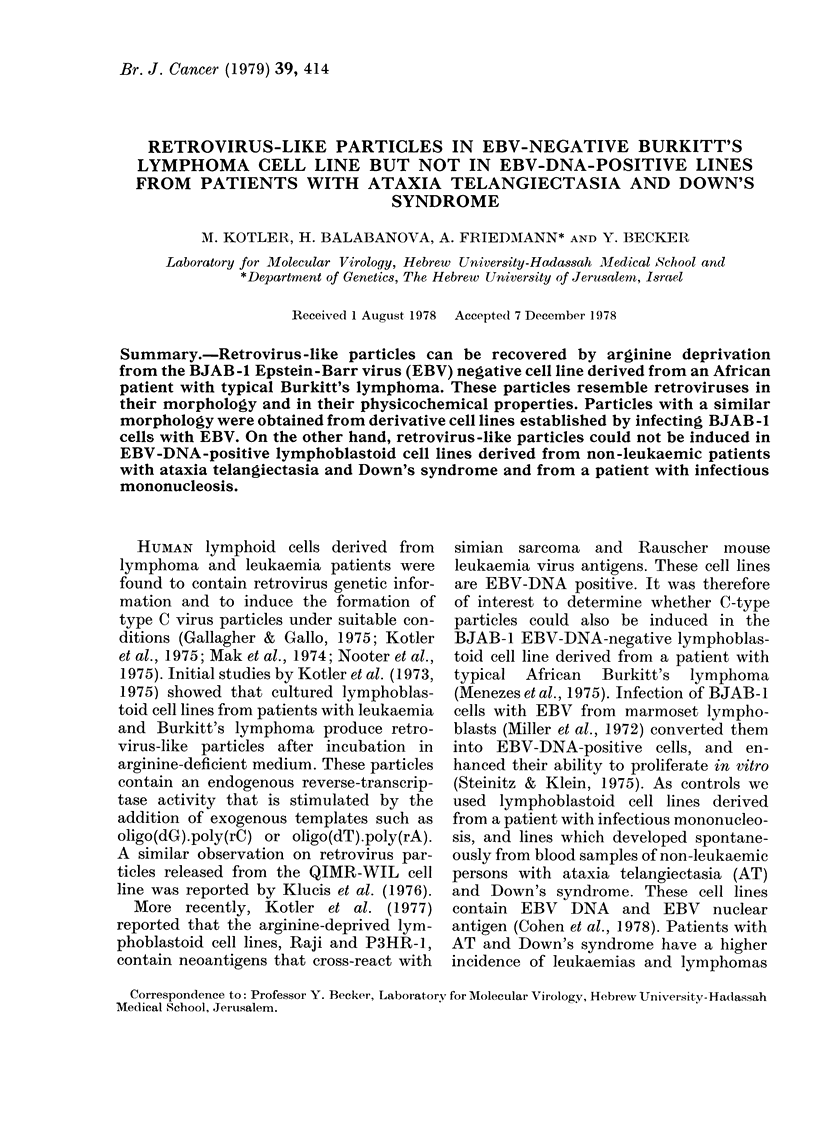

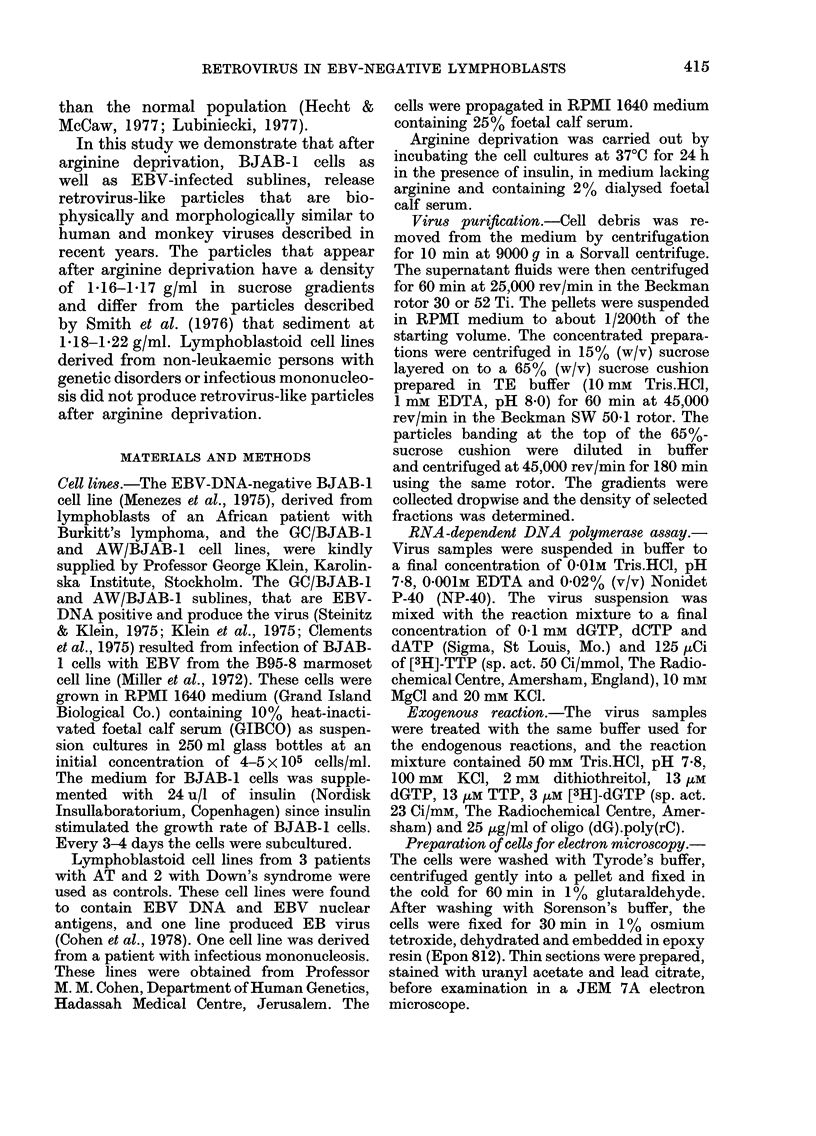

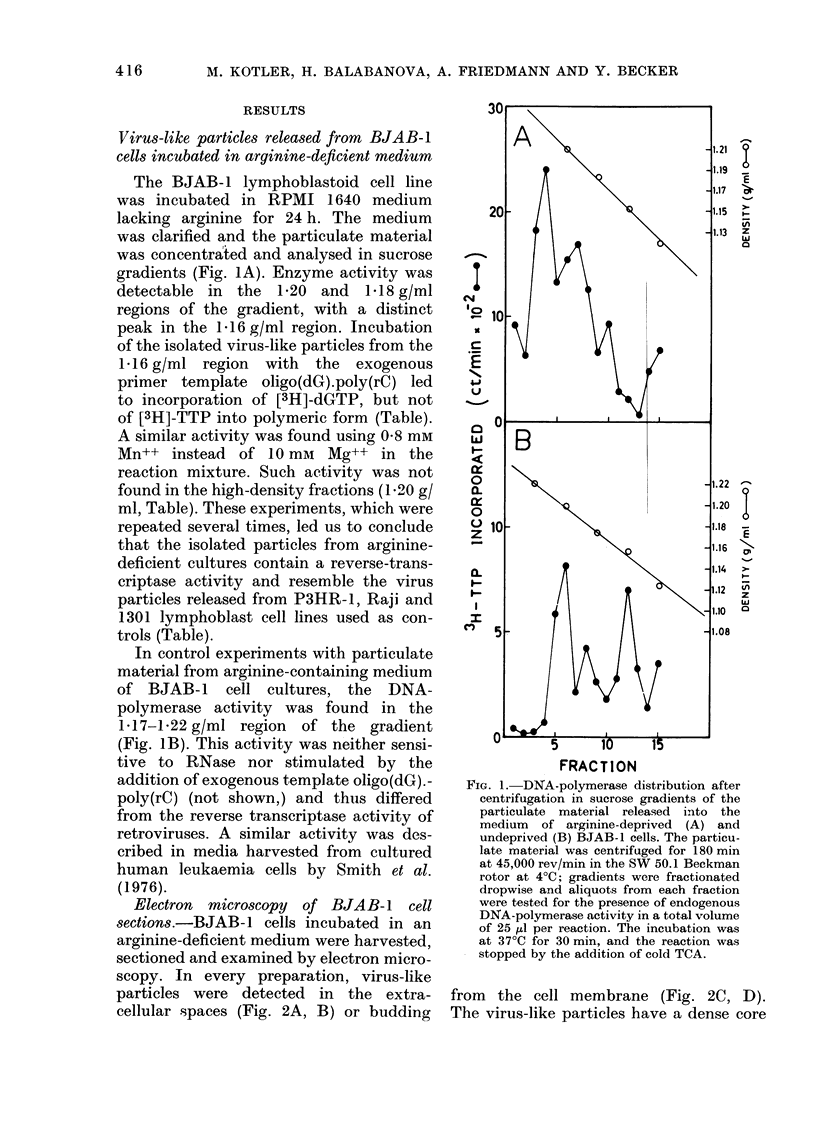

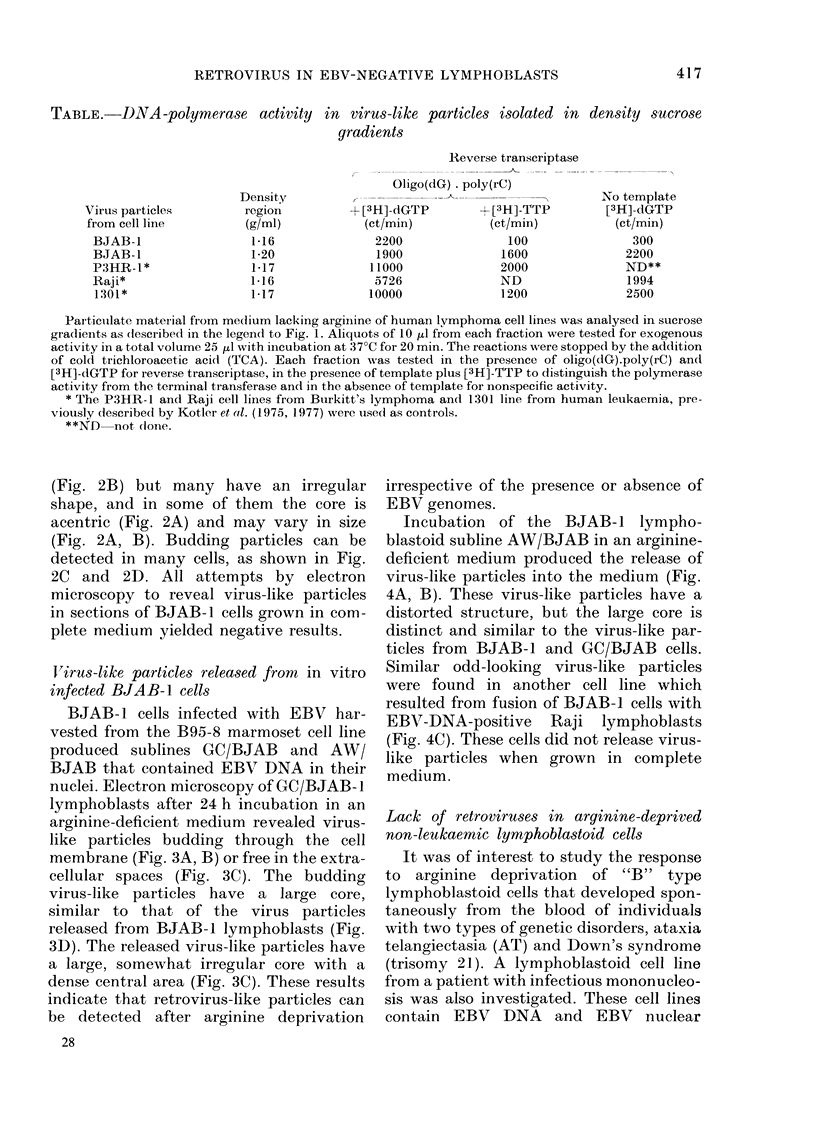

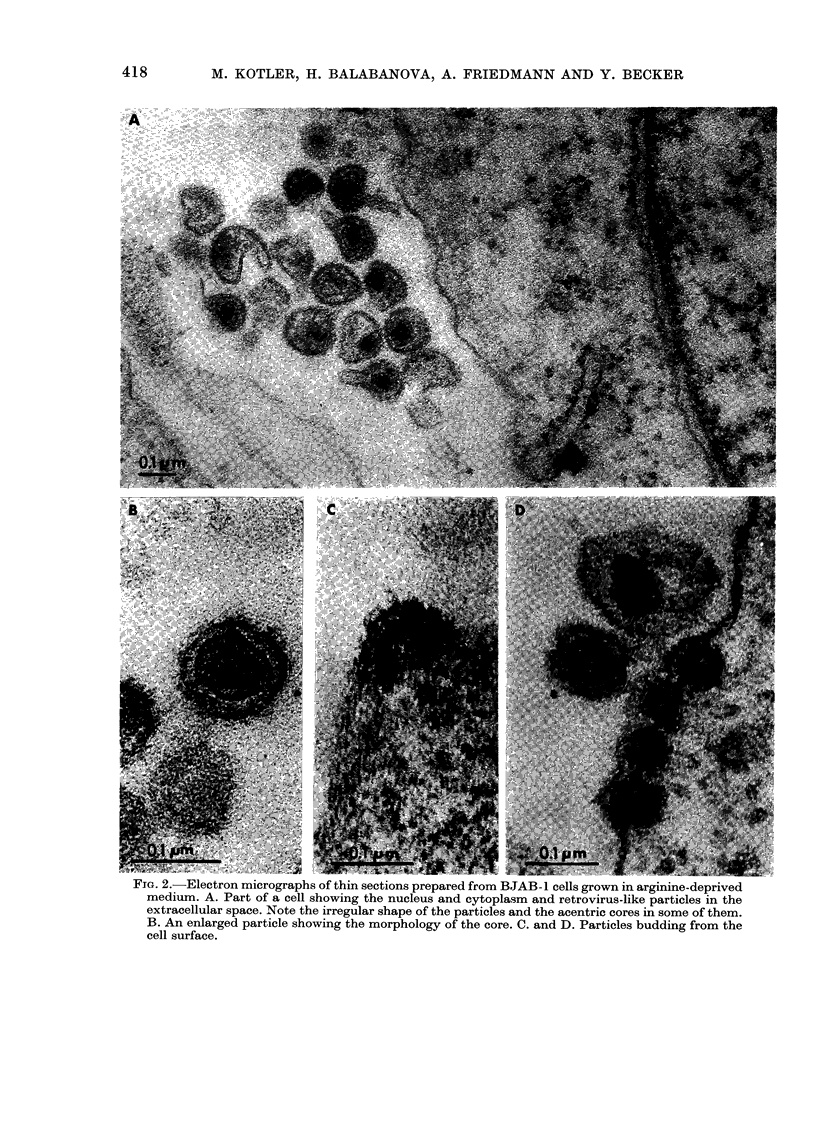

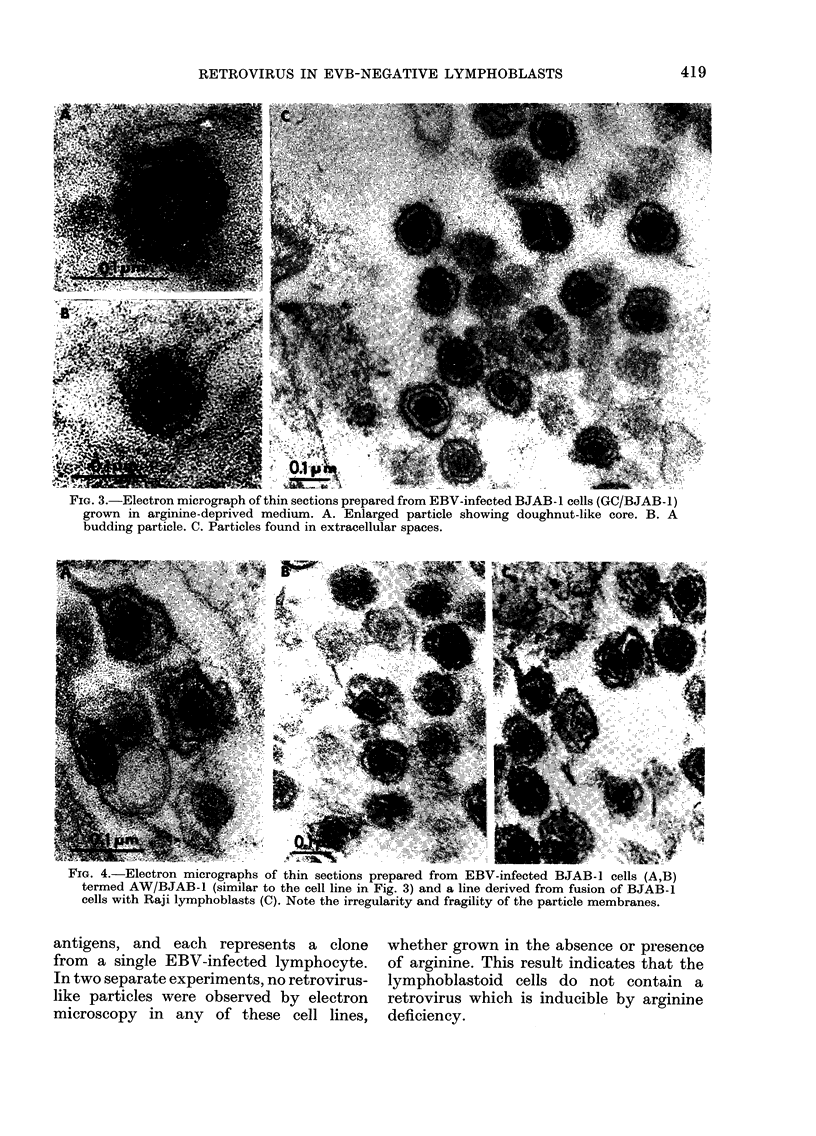

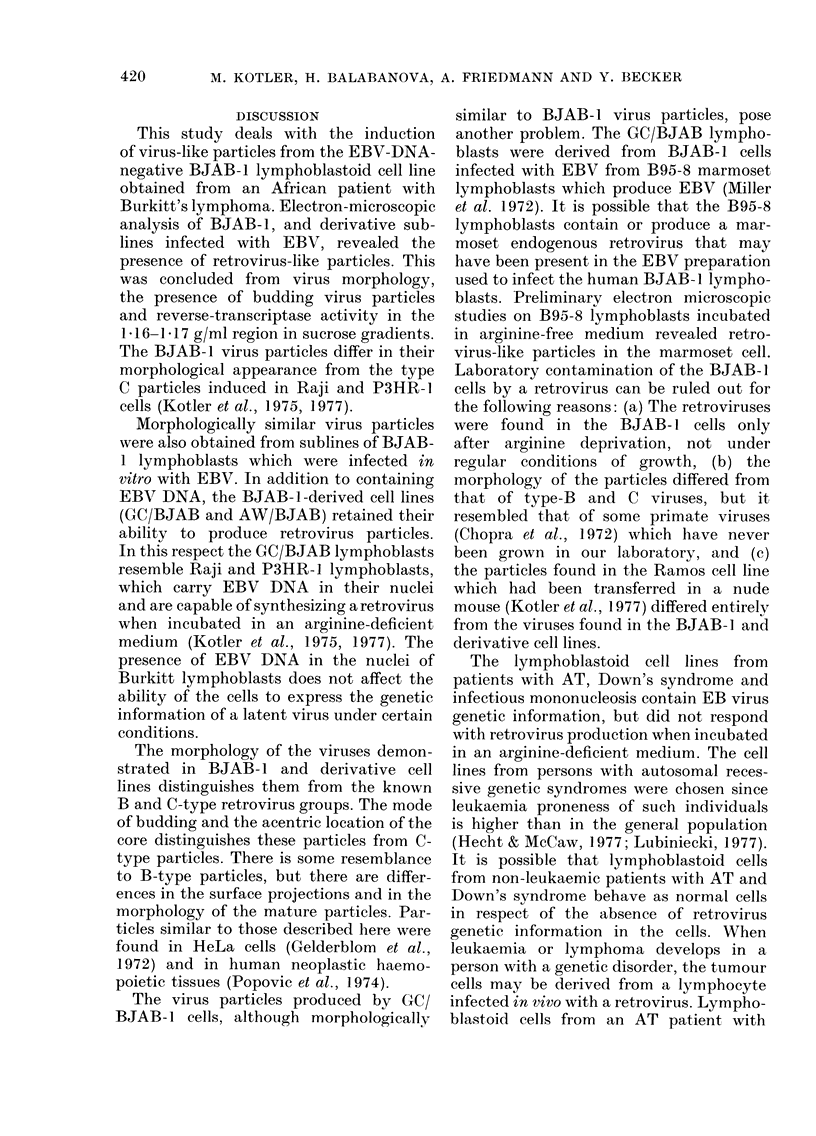

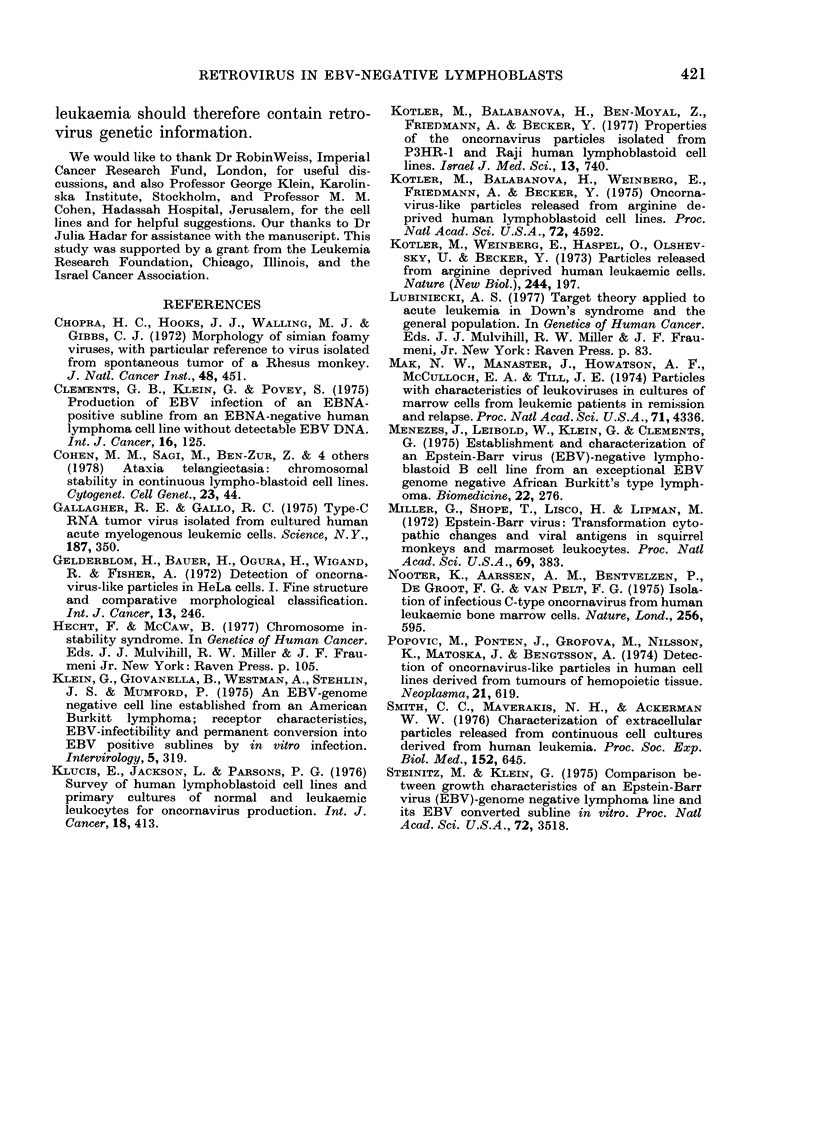


## References

[OCR_00666] Chopra H. C., Hooks J. J., Walling M. J., Gibbs C. J. (1972). Morphology of simian foamy viruses, with particular reference to virus isolated from spontaneous tumor of a rhesus monkey.. J Natl Cancer Inst.

[OCR_00673] Clements G. B., Klein G., Povey S. (1975). Production by EBV infection of an EBNA-positive subline from an EBNA-negative human lymphoma cell line without detectable EBV DNA.. Int J Cancer.

[OCR_00686] Gallagher R. E., Gallo R. C. (1975). Type C RNA tumor virus isolated from cultured human acute myelogenous leukemia cells.. Science.

[OCR_00692] Gelderblom H., Bauer H., Ogura H., Wigand R., Fischer A. B. (1974). Detection of oncornavirus-like particles in HeLa cells. I. Fine structure and comparative morphological classification.. Int J Cancer.

[OCR_00705] Klein G., Giovanella B., Westman A., Stehlin J. S., Mumford D. (1975). An EBV-genome-negative cell line established from an American Burkitt lymphoma; receptor characteristics. EBV infectibility and permanent conversion into EBV-positive sublines by in vitro infection.. Intervirology.

[OCR_00714] Klucis E., Jackson L., Parsons P. G. (1976). Survey of human lymphoblastoid cell lines and primary cultures of normal and leukaemic leukocytes for oncornavirus production.. Int J Cancer.

[OCR_00721] Kotler M., Balabanova H., Ben-Moyal Z., Friedman A., Becker Y. (1977). Properties of the oncornavirus particles isolated from P3HR-1 and Raji human lymphoblastoid cell lines.. Isr J Med Sci.

[OCR_00728] Kotler M., Balabanova H., Weinberg E., Friedmann A., Becker Y. (1975). Oncornavirus-like particles released from arginine-deprived human lymphoblastoid cell lines.. Proc Natl Acad Sci U S A.

[OCR_00735] Kotler M., Weinberg E., Haspel O., Olshevsky U., Becker Y. (1973). Particles released from arginine deprived human leukaemic cells.. Nat New Biol.

[OCR_00748] Mak T. W., Manaster J., Howatson A. F., McCulloch E. A., Till J. E. (1974). Particles with characteristics of leukoviruses in cultures of marrow cells from leukemic patients in remission and relapse.. Proc Natl Acad Sci U S A.

[OCR_00754] Menezes J., Leibold W., Klein G., Clements G. (1975). Establishment and characterization of an Epstein-Barr virus (EBC)-negative lymphoblastoid B cell line (BJA-B) from an exceptional, EBV-genome-negative African Burkitt's lymphoma.. Biomedicine.

[OCR_00762] Miller G., Shope T., Lisco H., Stitt D., Lipman M. (1972). Epstein-Barr virus: transformation, cytopathic changes, and viral antigens in squirrel monkey and marmoset leukocytes.. Proc Natl Acad Sci U S A.

[OCR_00769] Nooter K., Aarssen A. M., Bentvelzen P., De Groot F. G., Van Pelt F. G. (1975). Isolation of infectious C-type oncornavirus from human leukaemic bone marrow cells.. Nature.

[OCR_00776] Popovic M., Ponten J., Grofovå M., Nilsson K., Matoska J. (1974). Detection of oncornavirus-like particles in human cell lines derived from tumours of hemopoietic tissue.. Neoplasma.

[OCR_00783] Smith C. C., Maverakis N. H., Ackermann W. W. (1976). Characterization of extracellular particles released from continuous cell cultures derived from human leukemia.. Proc Soc Exp Biol Med.

[OCR_00790] Steinitz M., Klein G. (1975). Comparison between growth characteristics of an Epstein--Barr virus (EBV)-genome-negative lymphoma line and its EBV-converted subline in vitro.. Proc Natl Acad Sci U S A.

